# Hip resurfacing in a district general hospital: 6-year clinical results using the ReCap hip resurfacing system

**DOI:** 10.1186/1471-2474-13-247

**Published:** 2012-12-13

**Authors:** Walter van der Weegen, Henk J Hoekstra, Thea Sijbesma, Shennah Austen, Rudolf W Poolman

**Affiliations:** 1 Department of Orthopaedic Surgery, St. Anna Hospital, Bogardeind 2, Geldrop, EH, 5664, Netherlands; 2 Department of Orthopaedic Surgery, Onze Lieve Vrouwe Gasthuis, Amsterdam, The Netherlands

**Keywords:** Hip resurfacing, Implant survival, Adverse reaction to metal debris, ReCap design

## Abstract

**Background:**

The purpose of our study was to prospectively report the clinical results of 280 consecutive hips (240 patients) who received a ReCap Hip Resurfacing System implant (Biomet Inc., Warsaw, USA) in a single district general hospital. Literature reports a large variation in clinical results between different resurfacing designs and published results using this particular design are scarce.

**Methods:**

Mean follow up was 3.3 years (1.0 to 6.3) and four patients were lost to follow-up. All patients were diagnosed with end-stage hip osteoarthritis, their mean age was 54 years and 76.4% of all patients were male.

**Results:**

There were 16 revisions and four patients reported a Harris Hip Score <70 points at their latest follow up. There were no pending revisions. Kaplan-Meier implant survival probability, with revision for any reason as endpoint, was 93.5% at six years follow-up (95%-CI: 88.8-95.3). There were no revisions for Adverse Reactions to Metal Debris (ARMD) and no indications of ARMD in symptomatic non-revised patients, although diagnostics were limited to ultrasound scans.

**Conclusions:**

This independent series confirms that hip resurfacing is a demanding procedure, and that implant survival of the ReCap hip resurfacing system is on a critical level in our series. In non-revised patients, reported outcomes are generally excellent.

**Trial registration:**

ClinicalTrials.gov Identifier: NCT00603395

## Background

Hip resurfacing arthroplasty (HRA) has been widely used in recent years. Possible advantages of conserved femoral bone stock, low wear rates and low dislocation rates were the main reasons for surgeons to use HRA. Recent concerns on the use of Metal-on-Metal (MoM) bearings have intensified the discussion on HRA. The reported increase of metal ion levels after HRA with subsequent local Adverse Reactions to Metal Debris (ARMD) and poor results with revision for this complication have diminished the support for HRA [1-4].

In the published literature there is a wide range of clinical results between different HRA designs [3,5,6]. Although numeral clinical studies report short- and mid-term survival of different HRA systems, these studies focus on a limited number of HRA designs. To our knowledge, there are four studies published using the ReCap Hip Resurfacing System (Biomet Inc., Warsaw, USA). Gagala reported there were no significant complications after a maximum follow up (FU) of 20 months, using this implant design (n = 23) [7]. Baad-Hansen reported no significant translation or rotation using this implant design (n = 25), after two year FU using radiostereometry (RSA) [8]. A larger number of ReCap procedures (n = 137) with a three year FU are described in the Australian National Joint Replacement Registry. In this report a cumulative percent revision rate of 7.6% is presented for this specific HRA design [9]. Recently, Gross and Liu presented the mid-term results of 740 hip resurfacings with a 3.4% revision rate [10].

In this prospective study, we report the clinical results of 280 consecutive HRA’s using the ReCap Hip Resurfacing system, with a maximum FU of six years (range: 1–6). We hypothesised that implant survival would be compliant with the National Institute for Health and Clinical Excellence (NICE) benchmark (a revision rate of 10% or less at ten years, or consistent survival if only shorter FU is available) [11]. We further hypothesised that the risk for revision in subgroups based on gender, age and component size is comparable to findings in published literature.

## Methods

### Patients

Between September 2004 and September 2010 our first 280 consecutive, non-selected HRA procedures (240 patients) in a general district hospital were included in a prospective cohort study (Table 1).

**Table 1:** Demographics of the study group

	**Mean**	**Range**
Age at surgery (yr)	54	28 to 76
BMI	26.5	19 to 46
Hospital stay (days)	3.5	2 to 9
Follow up (months)	39	12 to 75
	Count	%
Sex (n = 240 patients)		
Males	187	77.9
Females	53	22.1
Diagnosis (n = 280 hips)		
Primary OA	258	92.1
DDH	19	6.8
Posttraumatic OA	3	1.1

OA indicates osteoarthritis; DDH, developmental dysplasia.

Patients diagnosed with end stage osteoarthritis (OA) were indicated for HRA. The entire group involved 240 patients (280 resurfacings) with a mean follow-up of 3.3 years (1 to 6.3) of whom 45 were followed-up for five years and 30 for six years.

Prior to surgery, a dual energy X-ray absorptiometry (DEXA) scan was made of all female patients and in all male patients suspected of osteoporosis. When T and Z values were below normal, patients were excluded from HRA. After informing the patient on the expected benefits and risks associated with HRA, informed consent on the surgery procedure and on study participations was obtained from all patients. Our study was approved by the Institutional Review Board. Patients with renal failure, femoral cysts, osteoporosis or a-vascular necrosis (AVN) of the femoral head were excluded. Female patients with a possible child wish were also excluded.

### Surgical technique and rehabilitation

**Figure 1:**
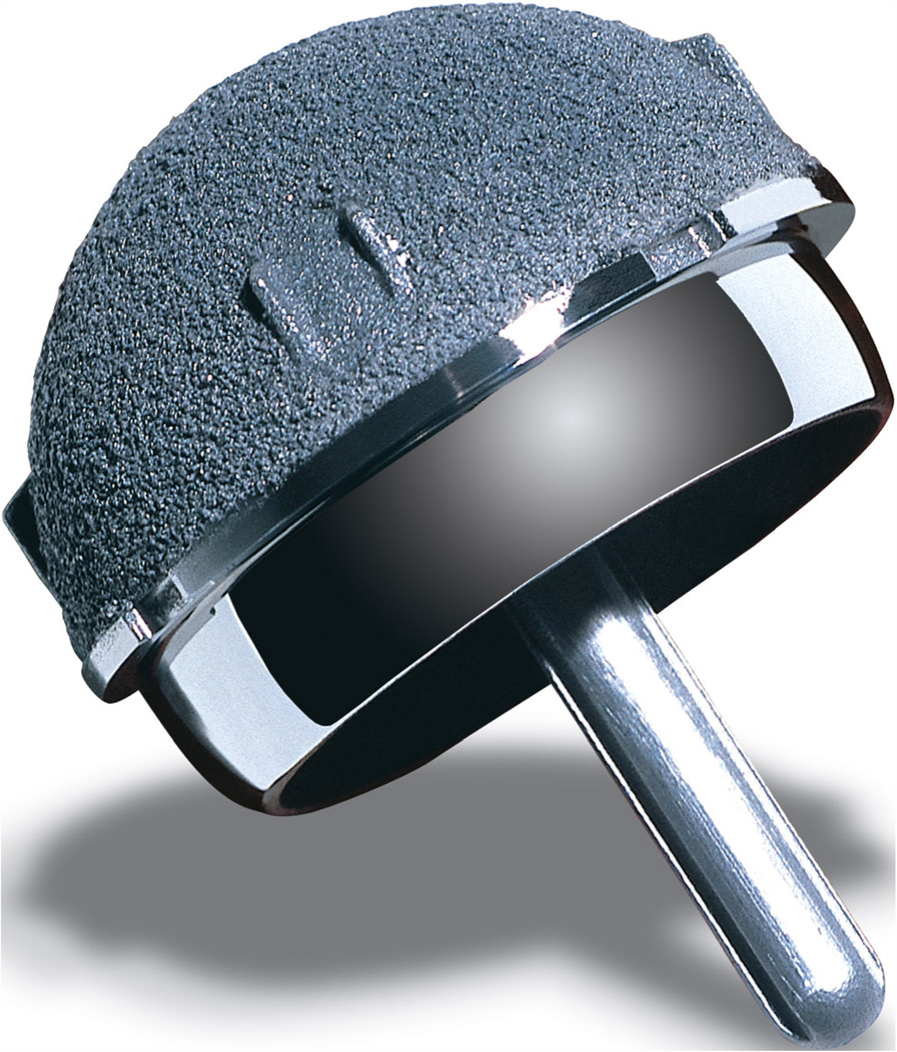
**ReCap resurfacing device** .

Two experienced joint arthroplasty surgeons (HJH, TS) used the ReCap Hip Resurfacing System (Biomet Inc, Warsaw, USA) in all patients in a standard manner. Prophylactic antibiotics were administered on induction. Both the press-fit acetabular component and the cemented femoral component are manufactured from “as-cast” cobalt chrome (Co-Cr-Mo) with a high carbon content (>0.2%). The acetabular outside is a full-hemisphere design and has four pairs of fins for initial rotational stability. It has a titanium porous plasma spray surface coating (Figure 1). The outer geometry of the cemented femoral component extends approximately 23 degrees beyond a full-hemisphere. The critical inner bearing surface has a coverage arc ranging from 155–164 degrees from smallest to largest component.

The posterolateral approach was used in all procedures. After dislocating the hip joint, acetabular osteophytes were removed, the acetabulum was reamed and the acetabular component was impacted into the anatomical position. Next, a femoral guide wire was inserted into the femoral head, directed with a jig. The femoral head was then circumferentially reamed and the bone-bed was prepared with drill holes and pulse lavage for cementing. After applying high viscosity cement (Refobacin® Bone Cement R, Biomet Europe, Dordrecht, Netherlands) to the inner surface, the femoral component was carefully put in place.

Patients were mobilised the first post-operative day using two crutches and weight bearing as tolerated. Patients were discharged if the patient was fully mobile and the wound was without problems. Physiotherapy was prescribed to all patients. Patients were instructed to avoid all high impact activities in the first six months and discouraged to participate in high impact sports. All bilateral procedures were staged interventions with at least a three months interval.

### Study protocol

Patients were recruited at the time of surgery and prospectively followed six weeks after surgery and yearly thereafter. Bilateral cases were followed up as separate cases. Standard antero-posterior (AP) and lateral radiographs, and the Harris Hip Score [12] were collected at each visit, except for the six week FU. Only radiographs were collected at this visit. Any patient who was symptomatic post-operatively was analysed with a diagnostic ultrasound scan to check for ARMD.

**Figure 2:**
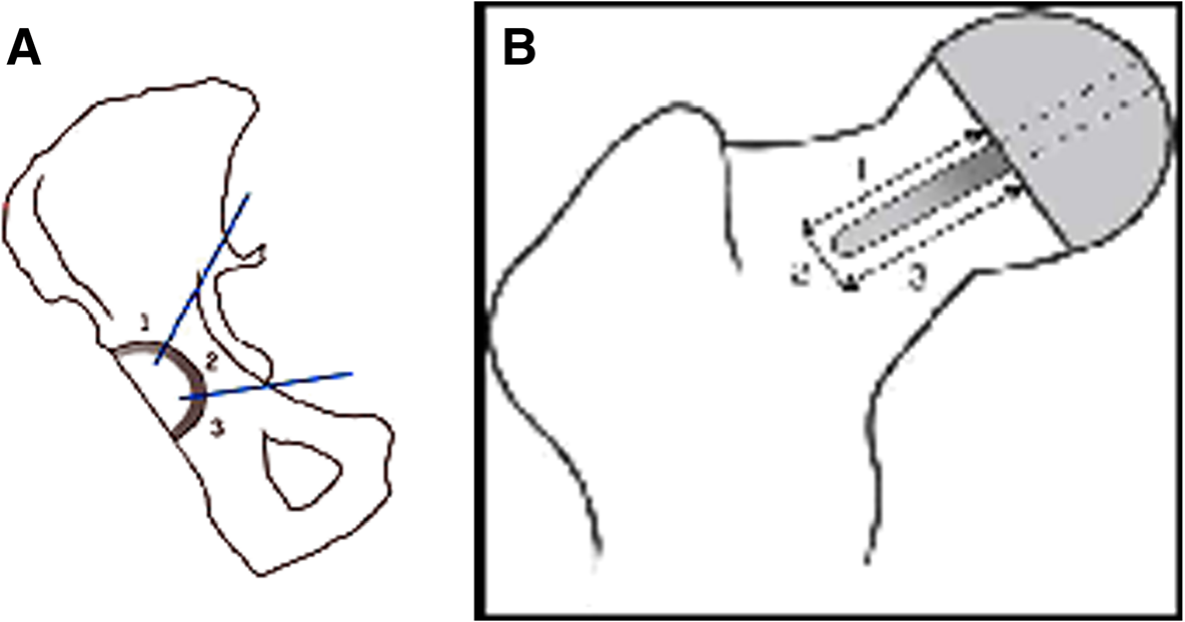
**A: Acetabular radiolucency zones according to DeLee and Charnley [**[14]**].B**: Femoral radiolucency zones according to Beaulé [13] .

On the plain anterio-posterior (AP) radiograph, the acetabular angle of inclination and femoral stem shaft angle were measured as described by Beaulé et al. [13]. Radiolucensies were measured in millimeters and acetabular radiolucency was classified in three zones according to DeLee and Charnley (Figure 2A) [14]. Any femoral radiolucencies were classified in the three zones as described by Beaulé et al. (Figure 2B) [13]. Heterotopic bone formation was classified as described by Brooker et al. [15]. Neck narrowing was measured as described by Grammatopoulos et al., using the first post operative radiograph and the most recent radiograph for comparison [16]. Clinical and radiological FU and statistical analyses were done by an independent observer, with a sample set of radiographic measurements audited by an experienced radiologist.

### Statistical analysis

Revision for any reason was the primary endpoint of this study. Kaplan-Meier survivorship curves were calculated. Since we support the recent notion in literature that implant survivorship is a limited endpoint to define a successful outcome for joint arthroplasty [17], a HHS score of < 70 points on the latest FU (two years or more) was also used as an endpoint for implant failure. The NICE benchmark (a revision rate of 10% or less at ten years, or consistent survival if only shorter FU is available) was used to evaluate survivorship [11]. Relative risks (RR) were calculated to evaluate sub-group results based on gender, age, component size and acetabular inclination angle. A femoral head size < 50 mm and an acetabular inclination angle of ≥ 55^0^ were considered to be a risk factor for ARMD and therefore revision. [18-20] SPSS software (SPSS Statistics, version 17.0, IBM Corporation, Somers USA) was used for all statistical analyses. The occurrence of femoral neck narrowing as a consequence to head downsizing can also be indicative for ARMD, as described by Grammatopoulos et al. [16]. Neck narrowing values were calculated as a percentage and ranges were presented for the whole cohort and for the patients who were revised > 6 months after index surgery.

## Results

Four patients were deceased for reasons not related to the HRA procedure (four prostheses, 1.4%) and no other patient was lost to FU. Three patients were contacted by phone since they were unable to return for FU. Therefore, radiological FU was complete for 277 patients. There were 16 revisions at the time of final FU. Seven were for fracture of the femoral neck, five for aseptic loosening of the acetabular component, two for component malpositioning (one femoral and one acetabular) and two for persistent pain (Table 2).

**Table 2:** Revision details

**Failure mode**	**Gender**	**Age**	**Fem.comp.**	**Months to revision**	** Revision details**
FN#	Male	61	48 mm	0.5	Femoral revision
FN#	Female	55	46 mm	0.5	Femoral revision
FN#	Male	57	52 mm	1	Femoral revision
FN#	Male	57	50 mm	1	Femoral revision
FN#	Male	60	52 mm	1	Femoral revision
FN#	Male	54	50 mm	2	Femoral revision
FN#	Male	48	48 mm	18	Femoral revision
Mal Fem Comp	Male	60	52 mm	0	Both comp. revised
Mal Acet Comp	Male	67	50 mm	12	THP other hospital
Asep Loosening	Male	58	54 mm	1	Both comp.revised
Asep Loosening	Male	64	50 mm	23	Both comp. revised
Asep Loosening	Female	49	44 mm	32	Both comp.revised
Asep Loosening	Male	28	50 mm	43	Both comp.revised
Asep Loosening	Female	49	42 mm	56	Both comp.revised
Persistent pain	Male	43	50 mm	7	THP other hospital
Persistent pain	Female	52	50 mm	27	Both comp.revised

FN# indicates fracture of the femoral neck; Mal Fem Comp: malpositioned femoral component; Mal Acet Com: malpositioned acetabular component; Asep Loosening: Aseptic loosening; Fem comp: femoral component size.

**Figure 3:**
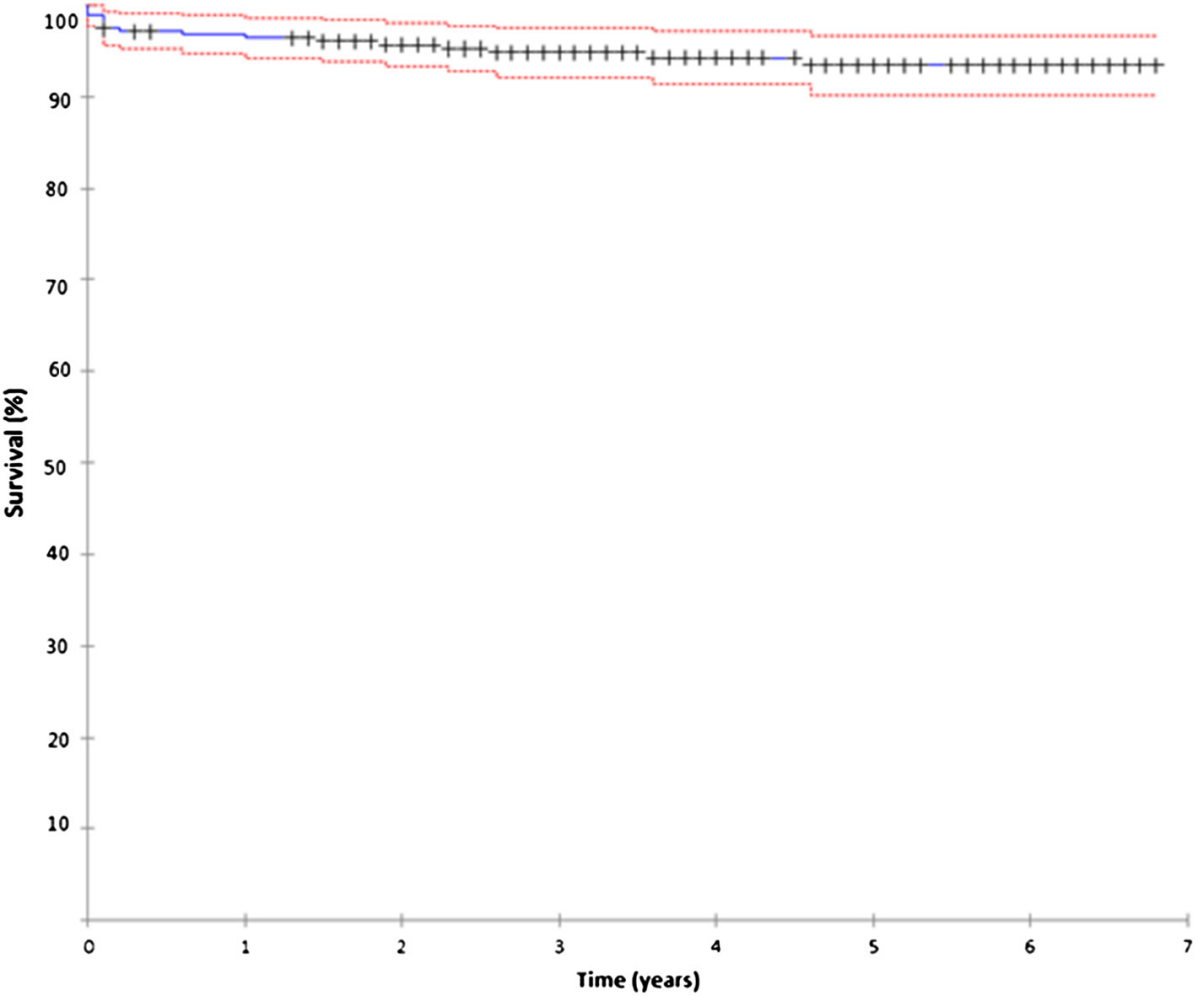
ReCap Kaplan-Meier implant survival probability

The Kaplan-Meier implant survival probability with revision for any reason as endpoint was 93.5% at six years FU (95%-CI: interval: 88.8-95.3) (Figure 3, table 3). The mean time to revision was 14 months (range: 0 – 56) with eight out of 16 revisions within two months from index surgery. Female patients had a RR for revision of 1.1 compared to male patients (95%-CI: 0.92-1.06). The RR for revision in the group of patients with a femoral head <50 mm, was 1.1 compared to the group of patients with larger components (95%-CI: 0.98-1.09). In the patients younger 55 years the RR for revision was 0.9 compared to patients 55 years or older (95%-CI: 0.95-1.07).

**Table 3:** Kaplan-Meier survival probability data details

** Year**	**Number of atstart**	**Failures**	**Withdrawn**	**Number of risk**	**Accumulated survival (%)**	**95% Confidence interval**
0 to 1	280	9	3	279.9	96.4	93.8 to 98.4
1 to 2	259	3	24	257.0	95.7	92.4 to 97.6
2 to 3	229	2	40	222.0	94.8	91.4 to 97.0
3 to 4	185	1	48	176.0	94.3	90.7 to 96.6
4 to 5	135	1	53	124.5	93.5	89.9 to 96.1
5 to 6	80	0	43	75.5	98.5	89.9 to 96.1
6 to 7	37	0	29	29.5	93.5	88.8 to 95.3

### Revision details

In all seven femoral neck fracture cases, the acetabular shell was left in situ and a stemmed, uncemented femoral prosthesis was inserted. Six out of seven neck fractures occurred within two months of the index surgery, one case was a late neck fracture 18 months post-operatively. During revision surgery of this one case it was observed that the femoral component was loose, which was thought to be caused by avascular necrosis of the femoral head. In all other cases both components were replaced. All cases of aseptic loosening only involved the uncemented acetabular component. Of the none-revised patients, there were four patients with a HHS score < 70 points at their latest FU (two at two years and two at three years FU). Revision and clinical score combined as endpoint for implant failure, resulted in 20 failed prostheses at the time of final FU. During revision surgery no metallosis, soft tissue cysts or solid masses were observed, although postoperative histopathological analyses showed chronic inflammatory signs including synovial hyperplasia en some metallosis in both patients revised for persistent pain, indicating adverse local tissue reaction to metal debris. A diagnostic ultrasound was made in 27 patients (9.6%) with unexplained hip or groin pains, all were normal. In our series there were 81 patients with an acetabular inclination angle of 55°-65° (of which 23 had a femoral head size <50 mm) and 10 patients with an acetabular inclination angle of >65° (of which four had a femoral head size <50 mm). In none of these patients any signs of ARMD were observed during any revision surgery or additional diagnostic ultrasound scans.

### Complications without need for revision

There were 30 (10.7%) complications without need for revision (Table 4).

**Table 4:** Complications without need for revision

**Complication**	**N (%)**
Nerve damage	2 (0.7%)
Non-displaced femoral neck fracture	2 (0.7%)
Deep wound infection	1 (0.4%)
Superficial wound infection	7 (2.5%)
Post-operative bleeding	18 (6.4%)
Total	30 (10.7%)

**Figure 4:**
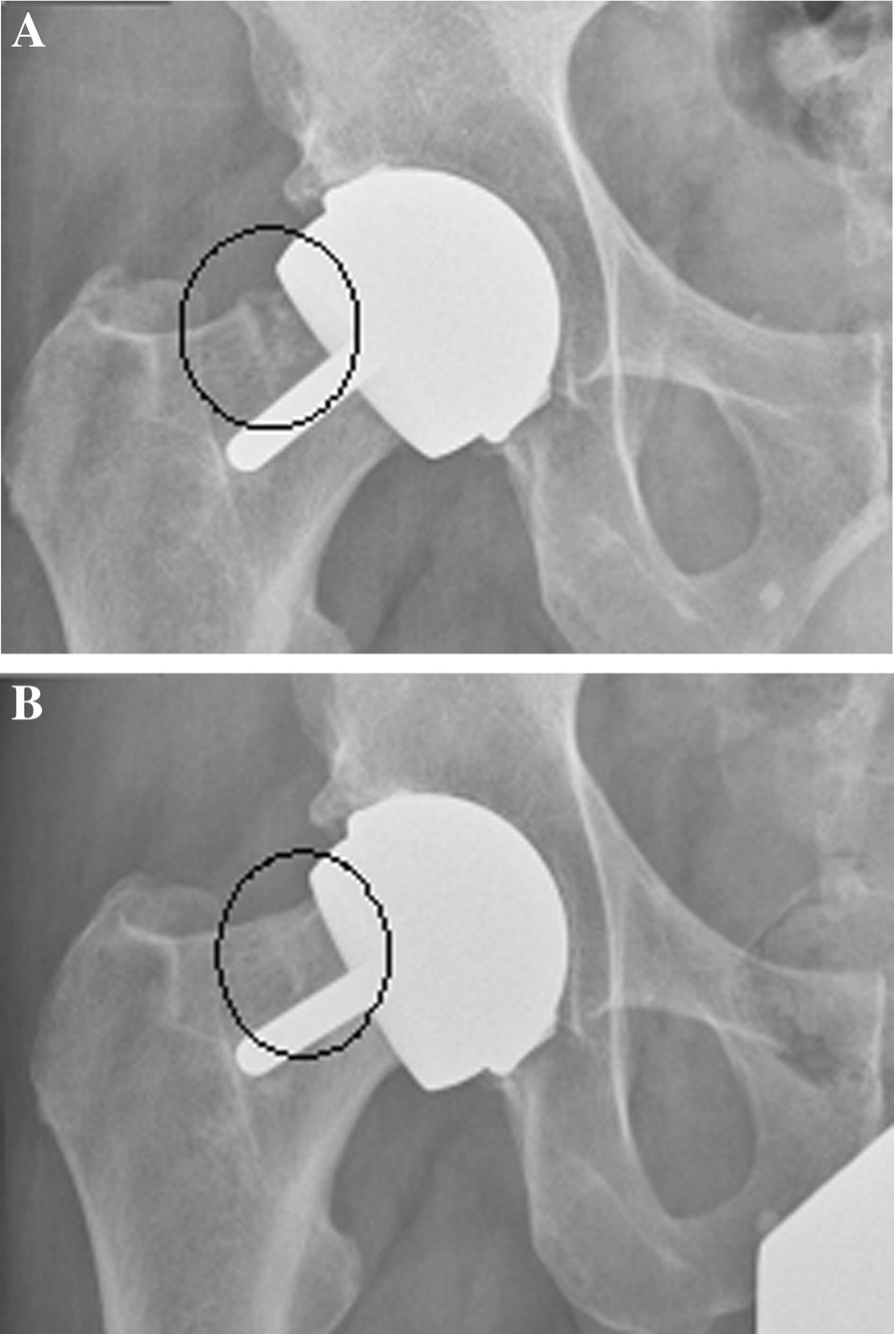
**A: Non-displaced partial femoral neck fracture.B**: Healed femoral neck fracture.

The majority of these complications were transient such as post operative bleeding (n = 18). There was one deep wound infection which was eradicated after surgical debridement and antibiotic treatment. Seven other patients with signs of a post-operative wound infection were treated successfully with antibiotics. There was one patient with persistent paraesthesia and pareses of the foot due to an sciatic nerve lesion. One other patient had a transient nerve palsy of the sciatic nerve. Another patient was treated conservatively for a non-displaced fracture of the femoral neck, which he sustained due to a fall three months after surgery. He recovered without any persistent symptoms. A healed non-displaced femoral neck stress fracture was discovered with routine FU two years post-operatively (Figure 4A and 4B). This patient had experienced some groin pain after running, which completely resolved when he did not run for a couple of weeks. There were no dislocations or thromboembolic events in our series.

### Outcomes

At one year FU, mean HHS had improved significantly from pre-operative scores (from 49.3 to 92, p < 0.0001, Table 5).

**Table 5:** Clinical and radiographic findings

	**HHS**	**Fem. Pos.**	**Cup abd. Angle**	**Brooker 1/2/3/4 (n)**
Pre op (n = 280)	49.3	n/a	n/a	n/a
6 wks (n = 280)	-	+2.2^0^	51.3^0^	13/2/0/0
1 yr (n = 280)	92	-	-	29/7/2/0
2 yrs (n = 221)	88.3	-	-	26/5/3/0
6 yrs (n = 54)	89.3	-	-	11/3/1/0

HHS indicates mean Harris Hip Score; Fem. Pos, mean varus/valgus placement compared to anatomical femoral neck angle (+ indicates varus position); Cup abd. Angle, mean cup abduction angle; Br. 1/2/3/4, Ectopic Bone Formation classified as Brooker grade 1, 2, 3,4; Radioluc., Radiolucensies.

At six year FU, 36 patients had an “excellent” HHS (66.7%), 16 a “good” HHS (29.6%) and two a “fair” HHS (3.7%). For the revised patients, the mean HHS after revision was 77 (range 41–91).

### Radiological findings

At one year FU, the mean implant femoral shaft angle was 135.1^0^ (range 116^0^-156^0^). Mean acetabular angle of inclination was 51.3^0^ (range: 26^0^ – 77^0^). With further FU, no radiolucensies were observed. Ectopic bone formation was noted in 13.8% of all cases. Mean HHS for patients who had a Brooker grade two or three ectopic bone formation was 91 points (range: 74–91) (Table 5). Neck narrowing was observed in 136 patients with a mean of 2.3% (range: 0%-18.5%). In the patients with revisions later than 6 months after index surgery, neck narrowing was present in 3 out of 9 patients. One patient had 2.5% neck narrowing and two patients had 6% neck narrowing.

## Discussion

Our KM-survival probability of 93.5% at six years FU (95%-CI: 88.8-95.3) is not compliant with the three year entry NICE benchmark. Longer FU is needed to compare our results with the full 10-year benchmark. Of the non-revised patients, there were only four patients with implant failure based on their HHS score. The combined endpoints of revision (n = 16) and HHS score < 70 points (n = 4), resulted in 20 failed prosthesis (7.1%). Since no other studies on MoM hip resurfacing have combined implant survival and Patient Reported Outcome scores to define implant performance, we cannot compare this result to other studies. We were able to identify all failure modes, including those from patients revised in other hospitals. Most frequent reasons for revision were fracture of the femoral neck (n = 7) and aseptic loosening (n = 5). All cases of aseptic loosening occurred relatively early and involved only the uncemented acetabular component. We think that insufficient seating of the acetabular component, which might occur due to deformation of the relatively thin cup during the impaction procedure, may have caused these early revision cases. In our series we have not observed any signs of ARMD during revision surgery, although post revision surgery two patients revised for persistent pain had histopathological evidence of adverse local tissue reaction (ALTR) to metal debris. Neither have we observed any signs of ARMD with diagnostic ultrasound scans in patients who were post-operatively symptomatic. We cannot completely rule out the presence of ARMD in our series, but since we observed two cases of ALTR, future follow-up will include routine metal ion analysis. Our complete FU, our detailed information on revision cases and the excellent clinical scores at the time of final FU are in contrast to other designs of HRA, of which failure rates of 25% for ARMD after six years FU are reported [21]. Risk factors for ARMD are the inclination angle of the acetabular cup, implant design, small component sizes and occurrence of neck narrowing. Steep inclination angles and an acetabular cup with less than hemispherical coverage result in a small contact patch area (CPA), which increases the wear rate. Another risk factor is component size, with small sizes resulting in more friction, releasing more metal debris [18-20]. In our series there were 81 patients with such risk factors, but no ARMD was observed in any of these patients, neither with a diagnostic ultrasound scan nor during revision surgery. The critical inner bearing surface of the ReCap has a coverage arc ranging from 155–164 degrees from smallest to largest component which is similar to other designs with a larger CPA such as the Birmingham Hip Resurfacing design (Smith and Nephew PLC, London, UK), the Conserve plus (Wright Medical Technology, Inc., Arlington, USA) and the Cormet resurfacing design (Corin Group PLC, Cirencester, UK). Our findings on ARMD are in line with several other studies. Malviya found a 0.15% incidence of pseudotumors using the Birmingham Hip Resurfacing (BHR) [22]. Beaulé et al. found a 0.1% prevalence of pseudotumors with MoM resurfacing after surveying nine Canadian Academic centers [23]. Glyn-Jones et al. extensively studied the risk factors for pseudotumor formation in a large series of hip resurfacings. Gender and age had a significant independent influence on the revision rate for pseudotumour formation, and the incidence increased with time, with a mean time to pseudotumour revision of 3.5 years (1 to 8.3 years) [24]. In the series presented by Steffen et al., there were three revision cases possibly related to metal debris. Two of these cases were revised around two years post-operatively, the other one at 5.6 years after surgery [25]. These mean times to pseudotumour revision are within the maximum follow-up time of our case series (6.3 years), but we will have to stay alert on ARMD occurrence with longer follow-up.

Grammatopoulos reported a mean 10.1% neck narrowing in patients revised for pseudotumours. In our cohort the mean percentage of neck narrowing was considerably lower (2.3%), although individual cases had greater neck narrowing. We did observe neck narrowing in three out of the nine patients who were revised > 6 months after index surgery, but these three patients had less than 10% neck narrowing. Neck narrowing data from our cohort is supplementary to the observations by Gross and Liu. They also report < 1% revisions for adverse wear and based on their report and on data from our cohort we believe that the risk for adverse wear using this resurfacing design is low. Gross did report a lower revision rate compared to our study (3.4% versus 7.3%) but in his series the learning curve was avoided since the surgeon had performed 400 hip resurfacings before the presented series was started [10]. As noted in the study by Gross, we also now have begun recommending routine metal ion tests in all our patients.

Strong points of our study are its prospective study design, a large consecutive study cohort, limited lost to FU and comparison to an objective benchmark. There is detailed FU on all revised patients including those revised in other hospitals, and both clinical outcome scores and radiological FU were analysed. Another advantage is that this study was conducted in a general district hospital rather than a design institution. Our study also has limitations: FU time is limited and there is no control group. We also have to bear in mind that the NICE-benchmark is applied to an OA population of all ages, and literature describes higher revision rates in younger patients [26-28]. Metal-ion levels were not obtained and there were no diagnostic ultrasounds made to check for ARMD in non-symptomatic patients. Compared to published literature, our study reports the clinical results on more patients with longer FU using the ReCap Hip Resurfacing system than any other study. Gagala et al. studied 25 patients (mean FU 11 months, range: 10–20) and found good short-term clinical results without significant complications [7]. Baad-Hansen et al. conducted a radiostereometric analysis (n = 23). There was no statistically significant translation or rotation of the femoral component observed after two years FU [8]. The absence of any revisions in these series might be due to the small number of patients and the short FU. In the evaluation of risk factors for early failure with HRA, the Australian Arthroplasty Register reported on 137 procedures between 1999 and 2008 using the ReCap hip resurfacing system [9]. Their cumulative percent revision rate of 7.6% at three year FU using this system was worse than our implant survival at three years. A possible explanation might be that those 137 procedures were done by a large number of orthopedic surgeons in an extended period of time, limiting the individual expertise using this system. However, despite further enquiry, no more details could be provided by the Australian Arthroplasty Register.

Regarding patient selection, in our series the RR for revision was slightly higher for female and for older patients, although statistically the difference was not significant. Patients with smaller component sizes had a higher risk for revision, but this was also not statistically significant. This is in line with several other publications which show a significantly higher risk for revision in female patients, older patients, and in patients with small components [5,29-32]. The possible absence of ARMD in our series might explain the equal risk for revision in patients with small or large component sizes. Looking at diagnosis, literature reports that the best HRA results are obtained with OA [5,33]. In our series, only patients with this diagnosis were included.

## Conclusion

Although implant survival rate in our series is below the NICE benchmark, patient reported outcomes are excellent in the non-revised patients. Also, we were not able to detect signs of ARMD with standard radiographs and clinical outcome scores. As with other resurfacing designs, this resurfacing system should be regarded as a difficult but effective surgical procedure for a small and specific patient population.

## Competing interests

WvdW, HJH and TS are supported by benefits directed to a research fund from Biomet Inc. SA declares no conflict of interest. RW P is supported by benefits directed to a research fund by Amgen, Lima, Link and Zimmer.

## Authors’ contributions

WvdW participated in the design of the study, data collection, analysis of the data, and drafting the manuscript. HJH participated in the design of the study, in data collection and drafting the manuscript. TS participated in the design of the study, in data collection and drafting the manuscript. SA participated in data collection and drafting the manuscript. RWP participated in drafting the manuscript. All authors read and approved the final manuscript.

## Pre-publication history

The pre-publication history for this paper can be accessed here:

http://www.biomedcentral.com/1471-2474/13/247/prepub
